# Psychometric Properties of the Revised Dysexecutive Questionnaire in a Non-clinical Population

**DOI:** 10.3389/fnhum.2022.767367

**Published:** 2022-03-02

**Authors:** Hannah Wakely, Ratko Radakovic, Andrew Bateman, Sara Simblett, Jessica Fish, Fergus Gracey

**Affiliations:** ^1^Faculty of Medicine and Health Sciences, Cambridgeshire and Peterborough NHS Foundation Trust, University of East Anglia, Norwich, United Kingdom; ^2^The Euan MacDonald Centre for Motor Neurone Disease, University of Edinburgh, Edinburgh, United Kingdom; ^3^Alzheimer Scotland Dementia Research Centre, University of Edinburgh, Edinburgh, United Kingdom; ^4^Centre for Cognitive Aging and Cognitive Epidemiology, University of Edinburgh, Edinburgh, United Kingdom; ^5^School of Health and Social Care, University of Essex, Colchester, United Kingdom; ^6^Department of Psychology, King’s College London, Institute of Psychiatry, Psychology, and Neuroscience, London, United Kingdom; ^7^Department of Clinical Neuropsychology and Clinical Health Psychology, St George’s Hospitals NHS Foundation Trust, London, United Kingdom; ^8^Mental Health and Wellbeing, Institute of Health and Wellbeing, University of Glasgow, Glasgow, United Kingdom

**Keywords:** dysexecutive problems, rating scales, validity, reliability, dysexecutive questionnaire-revised

## Abstract

**Aims:**

The aim of this study was to assess the psychometric properties of the revised self-rated version of the Dysexecutive Questionnaire (DEX-R) within a non-clinical sample.

**Methods:**

The study was hosted online, with 140 participants completing the DEX-R, GAD-2 and PHQ-2. Sixty participants also completed the FrSBe, with 99 additionally completing the DEX-R again 3 weeks later. Correlations with demographic factors and symptoms of anxiety and depression were conducted. Rasch and factor analysis were also used to explore underlying subconstructs.

**Results:**

The DEX-R correlated highly with the FrSBe, indicating sound concurrent validity. Internal consistency, split-half reliability and test-retest reliability were excellent. Age and symptoms of depression and anxiety correlated with DEX-R scores, with older age associated with less dysexecutive problems. The Rasch analysis confirmed the multidimensionality of the rating scale, and a three-factor structure was found relating to activation-self-regulatory, cognitive and social-emotional processes. Frequencies of responses on DEX-R items varied, many were not fully endorsed indicating specific relevance of most but not all items to patients.

**Conclusion:**

Interpretations of DEX-R ratings of dysexecutive problems should consider mood and individual variation. Systematic comparison of DEX-R responses between healthy and clinical groups could help identify a suitable cut off for dysexecutive symptoms.

## Introduction

There were an estimated 348,453 hospital admissions due to acquired brain injury (ABI) in 2016–2017 in the United Kingdom (Headway, 2018). ABI can arise through “trauma, vascular accident (e.g., stroke), cerebral anoxia, other toxic or metabolic insult (e.g., hypoglycemia), infection (e.g., encephalitis) or other inflammation (e.g., vasculitis)” (Turner-Stokes, 2003, p. 14) and can result in physical, cognitive, communication and emotional difficulties (Wilson et al., 2009). Those who have sustained a traumatic brain injury often present with difficulties associated with frontal lobe function (McDonald et al., 2002), due to the size, structure and location of the frontal lobes making them particularly vulnerable (Levin et al., 1987; Cicerone et al., 2006). Research on the structure and function of the frontal areas of the brain highlights roles in cognitive, behavioral and emotional processes. These include flexible thinking, planning, monitoring, social behavior, decision making, initiation, inhibition and emotional regulation (Lezak, 1995). The term “dysexecutive problems” has been used to describe difficulties with these functions, which can have a profound impact on a person’s level of independence resulting in challenges in day-to-day life (Hanks et al., 1999).

There have been challenges in the clinical measurement of dysexecutive problems, with various approaches to their assessment. Neuropsychological tests can be time-consuming and when used for assessment of frontal functions can be difficult to interpret and lack ecological validity due to the structure and presence of cues in the testing environment (Eslinger and Damasio, 1985; Damasio et al., 1991; Burgess et al., 1998). As a result, traditional tests often fail to highlight difficulties in this area despite reports of challenges in day-to-day life (Stuss et al., 1983; Shallice and Burgess, 1991). To overcome this, self-report measures have been developed to capture challenges faced in everyday life, which might complement neuropsychological assessments (Isquith et al., 2013). A limitation of using these with people experiencing dysexecutive problems is the issue of reduced self-awareness, meaning they may be more likely to underreport such difficulties (Simblett et al., 2017). Informant versions are available to corroborate or assess the discrepancy compared to the self-report version. Different rating scales have been developed and are available for clinical use, however, there are issues in the standardization and interpretation of scores. This is because the nature of these difficulties, such as decision making, perseveration and flexibility, could lead to issues with the reliability of item responses.

The Dysexecutive Questionnaire (DEX; Burgess et al., 1998) forms part of the Behavioral Assessment of the Dysexecutive Syndrome (BADS: Wilson et al., 1996). The DEX is a self-report measure of dysexecutive problems, designed to predict everyday difficulties. There are 20 items measuring behavioral, cognitive, motivational and emotional changes from pre-morbid functioning generating in a single score.

Simblett and Bateman (2011) assessed the psychometric properties of the DEX using item response theory by deploying Rasch analysis techniques. Their analysis suggested the DEX not to be a unidimensional measure of dysexecutive problems, instead capturing underlying sub-constructs thought to underpin these difficulties. Therefore, a total score on self-report measures may not best capture these challenges. In further research, Simblett et al. (2017) made amendments to the wording of some of the items in the DEX as well as including an additional 14 items to expand its measurement to incorporate Stuss’s (2011) proposed categories of frontal functions. After applying Rasch techniques, data from a clinical sample suggested the revised version of the DEX mapped onto the Stuss model capturing four separate sub-constructs of executive cognitive functions, metacognition, activation/energization and behavioral and emotional self-regulation. This enhances clinical application by enabling specific areas of strength and difficulty to be highlighted which can assist in diagnosis, neuropsychological formulation or become a focus for a person’s individual rehabilitation plan. The DEX-R has received further psychometric validation when applied to healthy aging and mental health samples (Loschiavo-Alvares et al., 2013; Dimitriadou et al., 2018) although factor analyses with these groups did not align with Stuss’s model. Instead, these studies produced a three-factor structure more in line with Fuster’s (2008) theory of frontal lobe functioning (Social Self-Regulation, Motivation and Attention, and Flexibility, Fluency and Working Memory).

Neurological disorders are known to contribute to reports of dysexecutive problems, however, additional understandings of how individual variation may manifest is useful for clinicians to understand. Research has highlighted individual variations in reported levels of dysexecutive problems in non-clinical populations as measured by the DEX questionnaire (Chan, 2001) due to individual differences or demographics such as age and mood or mental health. Normal aging processes have been associated with a decline in various cognitive functions associated with prefrontal areas (West, 1996; Van Petten et al., 2004). This may be more prominent in cognitive changes related to the dorsolateral regions, with less change from normal aging being found in ventromedial areas thought to underpin the emotional processing aspects of dysexecutive problems (MacPherson et al., 2002). The later maturation of ventromedial areas could have implications in the social and emotional functions being less developed in younger people (Burnett et al., 2009; Pfeifer et al., 2011; Sebastian et al., 2011; Barkley, 2012; Coffman, 2014). Negative affect may mediate the increased reports of dysexecutive problems in younger people (Gerstorf et al., 2008). Correlations have been found between dysexecutive and anxiety and depression symptoms, which may relate to cognitive variability in mood (Shaw et al., 2015). Another factor contributing to variation in reports of dysexecutive problems includes education level (Foss et al., 2013; Faria et al., 2015). The presence of these other factors influencing frontal functions presents a further challenge to the interpretation of clinical neuropsychological assessments.

It would therefore be useful to establish levels of reports of dysexecutive problems in non-clinical populations with questionnaire measures such as the DEX-R, to further explore psychometric properties and contribute toward the establishment of a normative level or cut off for identifying clinical levels of dysexecutive symptoms.

### Research Questions

The primary research questions were:

1. What is the factor structure of the DEX-R in a non-clinical population?
2. Does the DEX-R perform as an interval level measure as established by item response theory?
3. What are the measurement properties of the DEX-R within a non-clinical population?
  • 3a. Is the DEX-R a reliable measure of dysexecutive problems?
  • 3b. Is the DEX-R a valid measure of dysexecutive problems when compared to an existing valid self-report measure?

In addition, there were secondary research questions:

4. What are the effects of demographic and mood variables on DEX-R and DEX-R subscale performance?
4a. What are the effects of age on DEX-R and DEX-R subscale performance?

## Materials and Methods

### Participants

Participants aged 18 years or over were recruited into the study online via a snowball sampling recruitment method, whereby information about the study was distributed online through the research team’s networks, including social media. Recruitment took place from the 5th July 2019 to the 16th December 2019 where 140 people participated, of whom 99 completed the test-retest phase, and 60 the validity phase. Fifteen participants reported predefined health conditions, therefore group comparisons could not be made and they were excluded from the analysis. There were 125 participants included in the analysis (80% female) aged between 19 and 69 years (*M* = 37.7, *SD* = 12.6), 82% were educated to at least degree level and 77% were White British. See Table 1 for further demographic details.

**TABLE 1:** Demographic Information (*n* = 125).

	*n* (*%*)	Mean	*SD*
**Gender**			
Male	25 (20)		
Female	100 (80)		
Age		37.7	12.6
18–21	2 (2)		
22–28	28 (22)		
29–38	48 (38)		
39–55	33 (26)		
56–76	14 (11)		
76 +			
Years of education*		17.5	3.7
Up to 11 years	6 (5)		
12–14 years	21 (17)		
15–16 years	16 (13)		
17 + years	79 (65)		
**Highest level of education**			
Degree or equivalent	103 (82)		
Higher Education	4 (3)		
A Level or equivalent	7 (6)		
GCSE grades A*-C or equivalent	2 (2)		
Other qualification	9 (7)		
No qualification	0 (0)		
Other/Prefer not to say	0 (0)		
**Ethnicity**			
White (British)	97 (77)		
Other white background	15 (12)		
White (Irish)	4 (3)		
Indian	2 (2)		
Other Asian background	2 (2)		
Mixed White and Asian	2 (2)		
Other mixed background	1 (1)		
Black African	1 (1)		
Mixed White and Black ⋅ Caribbean	1 (1)		

* Three missing data for years of education. Source for categorizations, Office for National Statistics.

### Measures

#### Demographic Questions

Demographic questions included age, gender, highest education level, years of education and ethnicity.

#### Health Questions

Additional questions relating to health were included as part of the study to allow for monitoring whether clinical factors explained variance in the data, should this have arisen. These included: “Have you ever been formally diagnosed or hospitalized for the following conditions?,” and included neurodegenerative conditions (e.g., dementia, Parkinson’s disease, Huntington’s disease, Multiple sclerosis), neurodevelopmental conditions (e.g., autism spectrum disorder, attention deficit disorder (ADHD), learning disability), acquired brain injury, stroke, and mental health conditions (e.g., Bipolar disorder, Schizophrenia or Psychotic Illness). An “other” or “prefer not to answer” option was also available.

#### Dysexecutive Questionnaire-Revised

The DEX-R (Simblett and Bateman, 2011) is a 37-item questionnaire measuring dysexecutive problems which were developed using Rasch Analysis from the original DEX (Burgess et al., 1998). It is measured using a 5-point Likert scale, with response options of “Never,” “Occasionally,” “Sometimes,” “Fairly often” and “Very often,” coded from 0 to 4, respectively. Higher scores indicating greater reports of dysexecutive problems. It demonstrated good internal consistency reliability when applied with a clinical sample of people with ABI (Simblett et al., 2017). The four subscales of the DEX-R reflect the Stuss model: activation regulatory functions, behavioral-emotional self-regulatory functions, metacognitive functions and executive cognitive functions.

#### Frontal Systems Behavior Scale

The FrSBe (Grace and Malloy, 2001) is a 46-item self-report measure of dysexecutive problems which has been normed against non-clinical samples. Responses are coded as 1 (a*lmost never*) to 5 (*almost always*), with reverse scoring applied to a selection of items. Higher scores indicate more reported dysexecutive problems. It is composed of three sub-systems/subscales: executive dysfunction, apathy and disinhibition. The FrSBe demonstrates acceptable to excellent internal consistency reliability for total score and subscale scores ranging from Cronbach’s α 0.78–0.94 in neurological, mental health, non-clinical samples (Grace and Malloy, 2001; Velligan et al., 2002; Stout et al., 2003; Malloy and Grace, 2005). Construct validity and factor analysis supports the three factors of apathy, executive function and disinhibition thought to underpin the measure in various samples (Grace et al., 1999; Stout et al., 2003; Carvalho et al., 2013).

#### The Generalized Anxiety Disorder Scale

The Generalized Anxiety Disorder scale (GAD-2) is a 2-item, short, self-report measure of anxiety with a sensitivity of 65% and specificity of 88% for any anxiety disorder (Skapinakis, 2007). It is measured using a 4-point rating scale, with response options “Not at all,” “Several days,” “More than half the days” and “Nearly every day,” coded as 0 (*not at all*) to 3 (*nearly every day*), respectively. Higher scores suggest an increased presence of anxiety symptoms, with a clinical cut-off equal to or above three points (Skapinakis, 2007).

#### The Patient Health Questionnaire

The Patient Health Questionnaire (PHQ-2) is a 2-item, short, self-report measure with a sensitivity of 79% and specificity of 86% for detecting symptoms of depression (Löwe et al., 2005). It is measured using a 4-point rating scale, with response options “Not at all,” “Several days,” “More than half the days” and “Nearly every day,” coded as 0 (*not at all*) to 3 (*nearly every day*), respectively. Higher scores suggest an increased presence of depressive symptoms with a clinical cut-off with a score of three or above (Löwe et al., 2005).

### Procedure

The study was made available online via Qualtrics survey software (Snow and Mann, 2013). It was circulated online by the research team’s network. Participants were directed to the participant information sheet and could opt-in by providing an email address. A link to the study with a password for access was sent, this enabled access to a consent form. Participants were then directed to the DEX-R questionnaire, the FrSBe, PHQ-2, GAD-2, demographic questions and health questions. Participants could opt-in to complete the DEX-R again 3 weeks later.

### Ethical Considerations

The study was approved by the Faculty of Medicine and Health Sciences Research Ethics Committee at the University of East Anglia (UEA), reference number 201819–032. Participants gave informed consent and they were made aware of their right to withdraw by closing the survey.

### Analysis

Data cleaning was completed by removing incomplete responses. Parametric assumptions were checked using histograms and Shapiro-Wilk tests. Where item responses were not normally distributed transformations were attempted, otherwise non-parametric alternatives were used. Homogeneity of variance was checked using Levene’s test of equality of variance for *t*-tests. The study used these techniques as the removal of outliers would limit interpretations of non-clinical responses on the DEX-R, which could make comparisons with clinical groups difficult. To provide consistency in reporting, interpretations of psychometric properties were derived from the literature (Hermans et al., 2011).

Data were analyzed using the Statistical Package for the Social Sciences (SPSS), R, and RUMM 2020/2030.

**1. What is the factor structure of the DEX-R in a non-clinical population?**

Exploratory factor analysis (EFA) was used to establish the underlying structure and latent constructs of the DEX-R and whether this supports previous research with the DEX-R with a clinical population (Simblett et al., 2017). The decision on the number of factors to extract was determined by a parallel analysis using R software (Horn, 1965). It is recommended that an oblique rotation is first applied, and if the factor correlations are above 0.32 then this rotation is maintained (Pedhazur and Schmelkin, 1991; Tabachnick et al., 2007). SPSS was used to run the Principal Axis Factoring. It is recommended that factor loadings below 0.3 are suppressed (Field, 2013).

**2. Does the DEX-R perform as an interval level measure as established by item response theory?**

Rasch analysis was completed using the software RUMM2030 (Andrich et al., 2009). It is underpinned by item response theory which aims to calibrate both the difficulty of items as well as an individual’s ability. It establishes whether a questionnaire can be classed as an interval level measurement, as opposed to ordinal. Whether the DEX-R performs as a unidimensional measure was also explored with Rasch analysis because this identifies whether it is formed of subscales. If the chi-square value is not significant this confirms there is a misfit with the Rasch model, and therefore infers it is a unidimensional measure. If the data does differ significantly, this implies that the DEX-R is not a unidimensional measure, and therefore measuring multiple subconstructs. Multidimensionality was explored further by the factor analysis detailed in research question one.

**3. What are the measurement properties of the DEX-R within a non-clinical population?**

Internal consistency reliability of the DEX-R was assessed using Cronbach’s standardized α and split-half reliability which measure the consistency of the questionnaire to establish whether the questions relate to each other (Cronbach, 1951; Messick, 1989). A criticism of these are their lack of accountability for day-to-day variability. Therefore, test-retest reliability was also assessed using Intra Class Correlation (ICC) to measure whether the questionnaire is consistent over time. A 3-week interval for the test-retest phase was chosen in line with previous research (Cummings et al., 1994; Gioia et al., 2000; Holst and Thorell, 2018). A value of 0.7 or above is recommended to establish adequate reliability (Hermans et al., 2011).

Validity refers to whether the questionnaire actually measures what it sets out to, in this instance, whether the DEX-R measures dysexecutive problems (Messick, 1989). This was assessed through concurrent validation, by establishing if there was any correlation between the DEX-R and another validated measure of dysexecutive problems, the FrSBe. This was completed using Pearson product moment correlation coefficient.

**4**. **What are the effects of demographic and mood variables on DEX-R and DEX-R subscale performance?**

In order to determine whether demographic or mood variables are associated with variation in scores on the DEX-R, the study also compared subgroups (e.g., gender) and correlations with continuous variables (e.g., age) with total DEX-R scores and each DEX-R subscale. This used Pearson product moment correlation coefficient. These variables included age, gender, years of education, anxiety and depression scores. The GAD-2 and PHQ-2 were to be analyzed as a continuous measure unless a high proportion scored above the established cut off, in which case the groups were to be compared between those scoring above and below three. As multiple correlations were being used, the Bonferroni correction was applied with the alpha level set at 0.01 Regression analysis was used to identify which factors predict dysexecutive domains or total score. Gender was the only category variable and was converted into a binary variable. Non-parametric tests were used as the DEX-R, GAD-2, and PHQ-2 total scores were not normally distributed, and the latter were unable to reach normality via transformation. Descriptive statistics were used to explore the frequency of participant responses on the items of the DEX-R.

Separate sample size estimates were calculated for each question, the largest requirement being for correlation analyses, requiring at least 109 participants. This was calculated using G* Power 3.1.9 (Faul and Erdfelder, 1992), with power set at 0.9 to detect a medium effect size and probability was set at 0.05. This calculation was repeated for the multiple regression analysis with the addition of there being four variables included in the model, which indicated 82 participants were required for this analysis.

## Results

**1. What is the factor structure of the DEX-R in a non-clinical population?**

The parallel analysis retained three factors. The principal axis factoring using the oblique rotation resulted in appropriate correlations for this rotation method to be applied (Pedhazur and Schmelkin, 1991; Tabachnick et al., 2007). The factor analysis had adequate sampling (Kaiser-Meyer-Olkin: 0.83) and correlation (Bartlett’s Test of Sphericity: < 0.01). The factor loading matrix is presented in Table 2. The three factors accounted for 42.2% of the total variance, with the first factor accounting for 31.1%. Items with factor loadings below 0.3 were excluded from reporting. The internal consistency of factor one was excellent, good for factor two and questionable for the third factor (Hermans et al., 2011). As there were only three factors, the results, therefore, do not align with the Stuss model of frontal functions. The development of the DEX-R found some items did not map onto any of the subscales, these were retained due to their clinical utility (Simblett et al., 2017). Therefore, despite not all items achieving factor loadings above 0.4, no attempts were made to purify the model to preserve this utility.

**TABLE 2:** DEX-R items according to Stuss subscales and EFA with Promax rotation.

Item number	Stuss subscale according to Simblett et al. (2017)	Item description	Factor		
			1	2	3
3.	Activation	Apathy	**0.79**	−0.26	-0.04
26.	Metacognition	Inability to inhibit responses	**0.68**	−0.21	0.29
30.	Activation	Restlessness	**0.65**	−0.08	−0.08
11.	Activation	Perseveration	**0.64**	−0.15	0.27
32.	Behavioral-emotional self-regulation	Knowing doing dissociation	**0.64**	−0.11	0.18
22.	Previously metacognition, later removed	Cognitive control	**0.62**	0.23	−0.19
28.	Behavioral-emotional self-regulation	Emotional lability	**0.60**	0.07	−0.15
4.	Activation	Initiation	**0.59**	0.25	−0.13
19.	Behavioral-emotional self-regulation (previously Activation)	Insight	**0.57**	−0.01	0.28
10.	Metacognition	Anger	**0.53**	−0.01	-0.09
25.	Executive cognition	Organizational ability	**0.51**	0.17	−0.02
9.	Executive cognition	Verbal fluency	**0.47**	**0.30**	−0.20
24.	Behavioral-emotional self-regulation	Physical aggression	**0.46**	0.00	−0.08
23.	Activation	Variable motivation	**0.45**	**0.32**	−0.18
7.	Activation	Intention	**0.44**	**0.34**	−0.08
14.	Metacognition	Metaworry	**0.40**	0.29	−0.01
37.	Activation	Decision making	**0.40**	**0.38**	−0.08
6.	Metacognition	Social disinhibition	**0.34**	0.23	0.10
21.	Executive cognition	Temporal sequencing	**0.32**	0.15	0.25
27.	Behavioral-emotional self-regulation	Confabulation	0.28	0.06	−0.12
13.	Executive cognition	Abstract thinking	−0.10	**0.84**	−0.10
5.	Executive cognition	Planning	−0.07	**0.76**	-0.16
36.	Executive cognition	Complex attention	−0.08	**0.64**	0.05
31.	Metacognition	Cognitive confidence	−0.07	**0.61**	−0.07
17.	Executive cognition	Working memory	0.12	**0.56**	0.07
29.	Executive cognition	Distraction	0.19	**0.54**	0.01
20.	Behavioral-emotional self-regulation	Inertia	−0.09	**0.52**	0.16
33.	No subscale	Blunted affect 2	−0.10	**0.49**	**0.41**
2.	Previously executive cognition, later removed	Prospective memory	0.15	**0.48**	0.08
12.	Activation	Performance monitoring	−0.07	**0.36**	**0.33**
1.	Metacognition	Impulsivity	0.02	**0.33**	0.14
18.	Metacognition	Lack of social composure	0.11	**0.30**	0.29
35.	Metacognition	No concern for social rules	−0.20	0.01	**0.69**
15.	Behavioral-emotional self-regulation	Lack of concern	0.04	−0.11	**0.39**
16.	Behavioral-emotional self-regulation	Blunted affect 1	−0.06	**0.30**	**0.37**
8.	Behavioral-emotional self-regulation	Verbal aggression	−0.21	−0.05	**0.37**
34.	Executive cognition	Information processing	0.19	0.26	**0.37**
Eigenvalues			10.93	1.48	1.38
% of variance			31.12	5.69	5.14
Cronbach’s α			0.92	0.88	0.68

*Factor loadings > 0.30 are shown in bold.*

Seven of the items cross-loaded onto more than one factor. The 19 items loading onto factor one related to processes associated with the medial/dorsal domain. These items spanned across the proposed Stuss subscales, although mainly encompassed those from the activation subscale. These factors commonly share themes of initiation, maintenance and responsiveness, such as the ability to activate or inhibit a behavior or thought and was therefore labeled as “activation-self regulation.” The 17 items loading onto factor two appeared to relate to dorsolateral domains, typically these items represent cognitive dysexecutive symptoms such as planning, decision-making, abstract thinking, memory and attention. Although it was recognized that both blunted affect items additionally loaded onto this factor, and all seven cross-loadings involved this factor. The higher factor loadings mainly included those in the proposed executive cognition Stuss subscale. This factor was therefore labeled as “cognition.” Items loading onto factor three related to processes associated with the orbitofrontal areas, these also shared the blunted affect items. Additionally, more items corresponded to the Stuss behavioral-emotional self-regulation subscale. However, the items also appear to relate to social-self regulatory dysexecutive symptoms, therefore this factor was labeled “social-emotional.”

**2. Does the DEX-R perform as an interval level measure as established by item response theory?**

The responses on the DEX-R did not show fit to the Rasch model which suggests it measures more than one subconstruct [χ^2^(74, *N* = 140) = 205.54, *p* < 0.001]. Many of the questions showed disordered thresholds, and scale responses were not all endorsed on items, therefore it was not possible to confirm the interval nature of the scale as these have not yet arrived at a stable solution. Due to the small number in the clinical groups, we could not compare the level of endorsement or differential item functioning.

**3a. Is the DEX-R a reliable measure of dysexecutive problems?**

The DEX-R had excellent internal consistency with Cronbach’s α at 0.93 for time one and 0.94 for time two (Hermans et al., 2011). Cronbach’s α were also conducted to establish the level of consistency if each item were removed (see Table 3). Removal of specific items did not yield significant changes to the DEX-R reliability, with α ranging from 0.93 and 0.94. Cronbach’s α was 0.92 for factor one, 0.88 for factor two and 0.68 for the third factor. Split-half reliability was 0.93 for time one, and 0.94 for time two. A high degree of reliability was found between DEX-R scores on two-time points. The average measure ICC was 0.92 with a 95% confidence interval from 0.88 to 0.95 [*F*(88, 88) = 12.4, *p* < 0.001]. The median interval between the two phases were 23 days (interquartile range: 21–28 days). Table 3 displays scores given for the first phase of completion of the DEX-R.

**TABLE 3:** Descriptive statistics and internal consistency for DEX-R items and total score.

Item	Mean (*SD*)	Item-total correlation	Cronbach’s α if item deleted
1. Impulsivity	1.06 (0.81)	*r* = 0.38	0.93
2. Prospective memory	1.13 (0.96)	*r* = 0.60	0.93
3. Apathy	1.14 (0.93)	*r* = 0.46	0.93
4. Initiation	1.40 (1.10)	*r* = 0.68	0.93
5. Planning	0.90 (1.02)	*r* = 0.50	0.93
6. Social disinhibition	1.14 (0.90)	*r* = 0.56	0.93
7. Intention	1.38 (1.13)	*r* = 0.65	0.93
8. Verbal aggression	1.98 (0.89)	*r* = -0.02	0.94
9. Verbal fluency	1.38 (0.92)	*r* = 0.54	0.93
10. Anger	1.00 (0.86)	*r* = 0.43	0.93
11. Perseveration	0.56 (0.85)	*r* = 0.60	0.93
12. Performance monitoring	0.63 (0.64)	*r* = 0.43	0.93
13. Abstract thinking	0.62 (0.79)	*r* = 0.57	0.93
14. Metaworry	1.07 (1.08)	*r* = 0.60	0.93
15. Lack of concern	0.94 (1.05)	*r* = 0.17	0.93
16. Blunted affect 1	0.77 (0.91)	*r* = 0.41	0.93
17. Working memory	1.03 (0.96)	*r* = 0.63	0.93
18. Lack of social composure	1.03 (1.02)	*r* = 0.51	0.93
19. Insight	0.46 (0.81)	*r* = 0.65	0.93
20. Inertia	0.98 (0.98)	*r* = 0.47	0.93
21. Temporal sequencing	0.50 (0.79)	*r* = 0.57	0.93
22. Cognitive control	1.18 (1.04)	r = 0.65	0.93
23. Variable motivation	0.61 (0.85)	*r* = 0.57	0.93
24. Physical aggression	0.26 (0.66)	*r* = 0.38	0.93
25. Organizational ability	1.10 (1.09)	*r* = 0.58	0.93
26. Inability to inhibit responses	0.77 (0.85)	*r* = 0.59	0.93
27. Confabulation	0.14 (0.35)	*r* = 0.23	0.93
28. Emotional lability	0.37 (0.67)	*r* = 0.52	0.93
29. Distraction	1.33 (0.97)	*r* = 0.65	0.93
30. Restlessness	0.79 (0.81)	*r* = 0.47	0.93
31. Cognitive confidence	0.73 (0.76)	*r* = 0.42	0.93
32. Knowing doing dissociation	0.56 (0.77)	*r* = 0.57	0.93
33. Blunted affect 2	0.75 (0.85)	*r* = 0.57	0.93
34. Information processing	0.62 (0.90)	*r* = 0.61	0.93
35. No concern for social rules	0.59 (0.874)	*r* = 0.23	0.93
36. Complex attention	0.89 (0.84)	*r* = 0.50	0.93
37. Decision making	1.27 (1.09)	*r* = 0.64	0.93

**3b. Is the DEX-R a valid measure of dysexecutive problems when compared to an existing valid self-report measure?**

The DEX-R had good concurrent validity when compared to responses given on another validated measure of dysexecutive problems, the FrSBe (Grace and Malloy, 2001). Both the total scores on the DEX-R and FrSBe were first transformed to achieve adequate normality, the correlation between DEX-R and FrSBe was *r* = 0.83, *p* < 0.01.

4. **What are the effects of demographic and mood variables on DEX-R and DEX-R subscale performance? In particular, what are the effects of age on DEX-R and DEX-R subscale performance?**

Pearson product moment correlation coefficient analysis was conducted to establish any influence on participants reports of dysexecutive problems. Gender (*r* = −0.02, *p* = 0.836) and years of education (*r* = −0.52, *p* = *0.551*) were not significantly correlated to DEX-R total and factor scores (*p* > 0.05). No significant differences were found between males and females *t* (123) = −0.208, *p* = 0.84. A negative correlation was found between responses on the first DEX-R administration and age (*r* = −0.27, *p* = 0.002). Age significantly correlated with factor one, *r* = −0.34, *p* = <0.01 and factor two *r* = −0.24, *p* = <0.01, but not with factor three, *r* = 0.03, *p* = 0.76. A cut off of three as specified by the literature for the GAD-2 and PHQ-2 indicated 18% of participants scoring above the anxiety threshold, and 7% scoring above the depression threshold. Due to the uneven group sizes correlation analysis was used to preserve validity. Spearman Rho correlations was applied when analyzing the PHQ-2 and GAD-2. Anxiety scores correlated with dysexecutive problems, *r* = 0.45, *p* = <0.01. Depression scores were moderately correlated to dysexecutive problems, r = 0.58, *p* = <0.01. The scores on the GAD-2 significantly correlated with factor one, *r* = 0.51, *p* = <0.01 and factor two *r* = 0.47, *p* =< 0.01, but not with factor three, *r* = 0.16, *p* = 0.16. The scores on the PHQ-2 significantly correlated with all the factors, factor one, *r* = 0.57, *p* = <0.01, factor two *r* = 0.45, *p* = <0.01, and factor three, *r* = 0.25, *p* <0.01.

The effect of background variables on DEX-R responses was analyzed using a multiple regression, the DEX-R total score was the dependent variable, and age, gender, GAD-2 and PHQ-2 scores were the independent variables. In model one, age and gender were kept constant and explained 9.5% of the variance whereas in model two the additional inclusion of the GAD-2 and PHQ-2 scores explained 31.9%, *F*(4, 120) = 14.05, *p* <0.001. The results found gender not to significantly predict DEX-R scores (*p* >0.05). In the first model, DEX-R scores decreased by 0.44 for every year older a participant was, however, when the model also accounted for mood, this decreased to a reduction of 0.17 for every year older and was no longer a significant effect. Controlling for age, gender, anxiety and depression scores, the regression coefficient [*B* = 2.51, 95% *CI* (0.21, 4.81) *p* <0.05] for the GAD-2 indicates that for each increased score on the GAD-2, the total DEX-R score will increase by 2.51. Furthermore, within the same model, the regression coefficient [*B* = 5.28, 95% *CI* (2.67, 7.88) *p* <0.05] for the PHQ-2 indicates that for each increased score on the PHQ-2, the total DEX-R score will increase by 5.28.

## Discussion

The DEX-R was initially developed to map onto the Stuss model. Both the self and informant report versions have demonstrated validity and reliability in both acquired brain injury and healthy aging samples (Simblett et al., 2017; Dimitriadou et al., 2018). The current study adds to the literature regarding the robustness of the psychometric properties of the DEX-R by evidencing its stability and consistency over time. Additionally, it extends the previous literature by evidencing concurrent validity of the DEX-R with the FrSBe. Furthermore, its application with a non-clinical population provides some consideration for clinicians on how individual differences and mood may contribute to responses.

The individual variability of dysexecutive problems in a non-clinical population supports previous research (Chan, 2001) with age and mood found to significantly correlate with the DEX-R as has previously been found (Shaw et al., 2015; Dimitriadou et al., 2018). We found age to be negatively correlated with the DEX-R, indicating that older participants reported less dysexecutive problems. This is inconsistent with wider literature that cognitive functions associated with prefrontal areas decline with normal aging (West, 1996; Van Petten et al., 2004) but is consistent with the finding that aspects of emotion regulation improve with age (Phillips et al., 2008). However, this finding is not robust given the sample demographics, with the oldest participant being 69 years old and only 11% being over 56 years of age. The effect of age seemed to be removed when incorporating mood into the model, linking somewhat to previous research where negative affect mediated responses of dysexecutive problems reported by younger people (Gerstorf et al., 2008). These findings could be due to the younger age of the sample and explained by theories of brain maturation, with prefrontal areas developing into people’s early 30’s (Barkley, 2012; Coffman, 2014). The ventromedial areas of the prefrontal cortex are known to mature later than other regions and are implicated in social and emotional functions and may therefore be less developed in this sample (Gerstorf et al., 2008; Burnett et al., 2009; Pfeifer et al., 2011; Sebastian et al., 2011).

The Rasch analysis evidenced the DEX-R as being multidimensional, supporting its development to capture underlying subconstructs of dysexecutive problems (Simblett et al., 2017). A factor analysis found three factors representing activation: self-regulatory, cognitive and social-emotional functions. When compared to the proposed Stuss domains, the factors appeared to share some overlap with “activation,” “executive cognition” and “behavior-emotional self-regulation.” Those items corresponding with the “metacognition” subscale appeared to be equally distributed across the three factors. Despite the results not aligning fully with the Stuss model, the three factors do appear to have some overlap with theoretical conceptualizations of frontal lobe functioning, as found in prior research (Loschiavo-Alvares et al., 2013; Dimitriadou et al., 2018). These authors propose three factors—Social Self-Regulation, Motivation and Attention, and Flexibility, Fluency and Working Memory—in line with Fuster’s (2008) theory. Therefore, whilst the current study supports previous research on the DEX-R being multidimensional, and factor analysis yielded three factors, a confirmatory factor analysis would be required to confirm fit to either the Stuss or Fuster models.

All the subscales correlated with symptoms of depression, albeit only a weaker correlation for the social-emotional factor. Only the activation-self regulatory and cognition factors correlated with symptoms of anxiety. The social-emotional factor may therefore represent reduced emotional reactivity or neutrality, as those items loading onto it included blunted affect, a lack of concern and no concern for social rules. The cognitive factor correlations may be driven by executive cognitive factors associated with depression and anxiety, such as difficulties with problem-solving and decision making, and working memory (Watkins and Brown, 2002; Hofmann et al., 2011). The activation-self regulatory factor may correlate with symptoms of depression and anxiety as they account for components of apathy and a lack of motivation. Therefore, individual differences found in the study, particularly the different components forming factor one, may reflect an overlay with the cognitive symptoms of depression such as apathy. The issue in determining overlap and directionality of mood and dysexecutive problems is further complicated by the lack of diversity in the sample. The demographics of the sample are reported to experience a higher incidence of anxiety (Jenkins et al., 2020). It may be that particular items on the DEX-R are more likely to be rated as occurring more frequently in those experiencing symptoms of anxiety or depression, such as working memory, problem-solving and decision making as previously noted. This might account for why DEX-R scores increased by two for every GAD-2 score, and by five for every PHQ-2 score.

The differences in the underlying subscales and subconstructs found in the current study compared with previous findings on the DEX and DEX-R may relate in part to the sample characteristics as it is well understood that executive or frontal type difficulties are noted in a range of neurological, neurodevelopmental and mental health conditions. A comparison of the endorsement of items by different clinical groups through differential item functioning could provide insights into those items more likely endorsed by specific clinical groups.

### Strengths and Limitations

The main limitation of the current study is that of sample size. Although adequate for the reliability and validity statistics, larger samples are typically required for robust Rasch analysis. With regard to validation, we used an existing validated questionnaire which is not necessarily the gold standard, which in clinical studies would usually be a diagnostic measure. A further limitation of the current study is the highly educated and largely white and female sample which significantly limits generalizability. The lack of diversity in the sample poses limitations in terms of clinical extension and applicability of the current findings. Another limitation with sample diversity is that those who are naturally less well organized or less motivated would be less likely to sign up for the study (Stuss, 2011). Furthermore, the homogeneity of the sample means that it was not possible to investigate individual variation of reported dysexecutive problems relating to education level (Foss et al., 2013; Faria et al., 2015). The secondary research question relating to healthy aging was challenging to answer due to the small number of participants over the age of 60. The study would have benefited from including additional demographic questions on demographics, such as occupation and lifestyle (e.g., alcohol consumption and smoking status) as these can contribute to variability in neuropsychological abilities (Glass et al., 2009; Fisher et al., 2014).

The use of a survey to administer the rating scales may be influenced by survey bias due to the self-reporting of behavior, cognition and mood. To overcome this, participants were not required to give their personal details and by doing it online it was hoped this would reduce such bias. Given the potential that some aspects of natural variation in frontal or executive skills might impact completion of study tasks, an email reminder was used to prompt and remind participants. It is hoped that this reduced potential bias especially with regard to those completing the 3-week retest task.

Questions relating to health were included as part of the study to allow for monitoring whether clinical factors explained variance in the data, should this have arisen. There was only a small number of such responses, resulting in a predominantly non-clinical sample. However, there were insufficient responses from people declaring a clinical issue meaning comparison between groups was not possible.

### Clinical Implications

The main finding of clinical relevance was the presence of behaviors typically attributed to dysexecutive problems in clinical settings also occurring in a non-clinical sample. The factors in the current study appear to capture emotional processes potentially reflecting emotional regulatory processes (Salas et al., 2019). Furthermore, the correlations with depression scores may indicate the measurement of apathy. This individual variation has implications for clinicians in their interpretation of assessment scores, such as an awareness that this natural variation in scores on these items may have been present prior to clinical diagnosis. Clinically it would therefore be helpful to conduct assessment of mood alongside the administration of the DEX-R to aid interpretation.

### Future Research

Future research could consider a comparison of item responses of healthy and clinical groups via differential item functioning. Additional investigation via a Rasch analysis could establish subscales, which may highlight differences between groups such as whether awareness and metacognitive processes are more applicable to those with prefrontal brain injuries. Engaging with participants from different ethnic groups and a range of educational backgrounds would help improve the generalizability of findings. Finally, a larger-scale study with a more diverse sample should look to conduct confirmatory factor analyses in clinical or mixed (clinical and non-clinical) samples to substantiate the Stuss substructure or explore how the DEX-R maps onto other models of frontal functions.

## Conclusion

The current study supports the psychometric properties of the DEX-R as found in previous research, being both a valid and reliable measure of dysexecutive problems. The number of factors had similarities with two previous studies. There was individual variation in responses, influenced somewhat by mood and age. This may have implications for clinicians in their interpretation of DEX-R scores. Future research could consider comparing the responses of clinical and non-clinical groups.

## Data Availability Statement

The raw data supporting the conclusions of this article will be made available by the authors, without undue reservation.

## Ethics Statement

The study was approved by the Faculty of Medicine and Health Sciences Research Ethics Committee at the University of East Anglia (UEA), reference number 201819–032. The patients/participants provided their written informed consent to participate in this study.

## Author Contributions

HW completed this research as part of a doctoral thesis, and was supervised by FG and RR. RR also supported part of the analysis phase using R. FG and RR contributed through regular meetings, guidance on analysis, and editing of the manuscript. AB, SS, and JF were involved in the initial discussions of the topic for the manuscript, advice on analysis, and editing. AB supported the Rasch analysis (RUMM). All authors contributed to the article and approved the submitted version.

## Conflict of Interest

The authors declare that the research was conducted in the absence of any commercial or financial relationships that could be construed as a potential conflict of interest.

## Publisher’s Note

All claims expressed in this article are solely those of the authors and do not necessarily represent those of their affiliated organizations, or those of the publisher, the editors and the reviewers. Any product that may be evaluated in this article, or claim that may be made by its manufacturer, is not guaranteed or endorsed by the publisher.
