# Supplementary material for: Psychometric Properties of the Revised Dysexecutive Questionnaire in a Non-clinical Population
Source: Front Hum Neurosci. 2022 Mar 2;16:767367. doi: 10.3389/fnhum.2022.767367 (PMC8924056; doi:10.3389/fnhum.2022.767367)
Supplement: Supplementary file 1 [file Table_1.docx]

**DEX-R item distribution for time 1.**

|  | | | | | |
| --- | --- | --- | --- | --- | --- |
| **Item Number and Description** | **Frequency (%)**  **(n = 125)** | | | | |
|  | **Never** | **Occasionally** | **Sometimes** | **Fairly often** | **Very often** |
| 1. Impulsivity | 32 (25.6) | 59 (47.2) | 29 (23.2) | 5 (4.0) | 0 (0) |
| 2. Prospective memory | 32 (25.6) | 59 (47.2) | 24 (19.2) | 6 (4.8) | 4 (3.2) |
| 3. Apathy | 32 (25.6) | 57 (45.6) | 24 (19.2) | 11 (8.8) | 1 (0.8) |
| 4. Initiation | 21 (16.8) | 56 (44.8) | 29 (23.2) | 15 (12.0) | 4 (3.2) |
| 5. Planning | 54 (43.2) | 43 (34.4) | 17 (13.6) | 8 (6.4) | 3 (2.4) |
| 6. Social disinhibition | 28 (22.4) | 65 (52.0) | 21 (16.8) | 9 (7.2) | 2 (1.6) |
| 7. Intention | 29 (23.2) | 49 (39.2) | 22 (16.6) | 20 (16.0) | 5 (4.0) |
| 8. Verbal aggression | 3 (2.4) | 38 (30.4) | 47 (37.6) | 33 (26.4) | 4 (3.2) |
| 9. Verbal fluency | 19 (15.2) | 56 (44.8) | 35 (28.0) | 13 (10.4) | 2 (1.6) |
| 10. Anger | 36 (28.8) | 62 (49.6) | 19 (15.2) | 7 (5.6) | 1 (0.8) |
| 11. Perseveration | 77 (61.6) | 32 (25.6) | 11 (8.8) | 4 (3.2) | 1 (0.8) |
| 12. Performance monitoring | 57 (45.6) | 57 (45.6) | 11 (8.8) | 0 (0) | 0 (0) |
| 13. Abstract thinking | 66 (52.8) | 45 (36.0) | 9 (7.2) | 5 (4.0) | 0 (0) |
| 14. Metaworry | 44 (35.2) | 48 (38.4) | 17 (13.6) | 12 (9.6) | 4 (3.2) |
| 15. Lack of concern | 57 (45.6) | 34 (27.2) | 20 (16.0) | 13 (10.4) | 1 (0.8) |
| 16. Blunted affect 1 | 60 (48.0) | 42 (33.6) | 16 (12.8) | 6 (4.8) | 1 (0.8) |
| 17. Working memory | 40 (32.0) | 53 (42.4) | 23 (18.4) | 6 (4.8) | 3 (2.4) |
| 18. Lack of social composure | 46 (36.8) | 43 (34.4) | 25 (20.0) | 8 (6.4) | 3 (2.4) |
| 19. Insight | 86 (68.8) | 27 (21.6) | 7 (5.6) | 4 (3.2) | 1 (0.8) |
| 20. Inertia | 44 (35.2) | 54 (43.2) | 16 (12.8) | 8 (6.4) | 3 (2.4) |
| 21. Temporal sequencing | 80 (64.0) | 32 (25.6) | 8 (6.4) | 5 (4) | 0 (0) |
| 22. Cognitive control | 33 (26.4) | 59 (47.2) | 14 (11.2) | 16 (12.8) | 3 (2.4) |
| 23. Variable motivation | 72 (57.6) | 36 (28.8) | 12 (9.6) | 4 (3.2) | 1 (0.8) |
| 24. Physical aggression | 102 (81.6) | 17 (13.6) | 3 (2.4) | 2 (1.6) | 1 (0.8) |
| 25. Organisational ability | 45 (36.0) | 42 (33.6) | 21 (16.8) | 14 (11.2) | 3 (2.4) |
| 26. Inability to inhibit responses | 53 (42.4) | 57 (45.6) | 7 (5.6) | 7 (5.6) | 1 (0.8) |
| 27. Confabulation | 107 (85.6) | 18 (14.4) | 0 (0) | 0 (0) | 0 (0) |
| 28. Emotional lability | 91 (72.8) | 23 (18.4) | 10 (8.0) | 1 (0.8) | 0 (0) |
| 29. Distraction | 17 (13.6) | 73 (58.4) | 17 (13.6) | 13 (10.4) | 5 (4.0) |
| 30. Restlessness | 52 (41.6) | 51 (40.8) | 18 (14.4) | 4 (3.2) | 0 (0) |
| 31. Cognitive confidence | 53 (42.4) | 56 (44.8) | 14 (11.2) | 1 (0.8) | 1 (0.8) |
| 32. Knowing doing dissociation | 68 (54.4) | 46 (36.8) | 9 (7.2) | 2 (1.6) | 0 (0) |
| 33. Blunted affect 2 | 57 (45.6) | 49 (39.2) | 12 (9.6) | 7 (5.6) | 0 (0) |
| 34. Information processing | 76 (60.8) | 28 (22.4) | 14 (11.2) | 7 (5.6) | 0 (0) |
| 35. No concern for social rules | 75 (60.0) | 31 (24.8) | 14 (11.2) | 5 (4.0) | 0 (0) |
| 36. Complex attention | 44 (35.2) | 59 (47.2) | 14 (11.2) | 8 (6.4) | 0 (0) |
| 37. Decision making | 31 (24.8) | 54 (43.2) | 20 (16.0) | 15 (12.0) | 5 (4.0) |
